# Informational architecture across non-living and living collectives

**DOI:** 10.1007/s12064-020-00331-5

**Published:** 2021-02-02

**Authors:** Hyunju Kim, Gabriele Valentini, Jake Hanson, Sara Imari Walker

**Affiliations:** 1 Beyond Center for Fundamental Concepts in Science, Arizona State University, Tempe, AZ USA; 2 School of Earth and Space Exploration, Arizona State University, Tempe, AZ USA; 3 ASU-SFI Center for Biosocial Complex Systems, Arizona State University and Santa Fe Institute, Tempe, USA; 4 School of Life Sciences, Arizona State University, Tempe, AZ USA; 5 Santa Fe Institute, Santa Fe, NM USA

**Keywords:** Information theory, Biological information, Living collectives, Collective behavior

## Abstract

Collective behavior is widely regarded as a hallmark property of living and intelligent systems. Yet, many examples are known of simple physical systems that are not alive, which nonetheless display collective behavior too, prompting simple physical models to often be adopted to explain living collective behaviors. To understand collective behavior as it occurs in living examples, it is important to determine whether or not there exist fundamental differences in how non-living and living systems act collectively, as well as the limits of the intuition that can be built from simpler, physical examples in explaining biological phenomenon. Here, we propose a framework for comparing non-living and living collectives as a continuum based on their information architecture: that is, how information is stored and processed across different degrees of freedom. We review diverse examples of collective phenomena, characterized from an information-theoretic perspective, and offer views on future directions for quantifying living collective behaviors based on their informational structure.

## Introduction

The broad class of phenomena we nominally refer to as “life” has so far proven elusive to concrete scientific characterization. While many definitions for life have been proposed (Cleland and Chyba 2002; Tirard et al. 2010; Benner 2010; Walker and Davies 2013), these are almost exclusively descriptive and not quantitative. Artificial life and systems chemistry approaches are pushing us ever closer to realizing “life-like” systems in the laboratory. Our efforts to find life on other worlds are also accelerating pace, driven in large part by discoveries of dozens of habitable worlds orbiting other stars (Kopparapu et al. 2014; Dressing and Charbonneau 2015; Anglada-Escudé et al. 2012, 2016; Gillon et al. 2017) and newly funded missions such as Dragonfly, which will visit Titan and may even search for signs of life.

In the absence of quantitative metrics to determine whether or not whatever it is we discover is indeed “life,” our search for new examples is left largely unguided. It is becoming increasingly imperative to develop rigorous, quantitative approaches to characterize what life is. Most efforts adopt a phenomenological approach to defining life’s most important attributes, for example citing replication, metabolic activity, the capacity to evolve, a cellular boundary, etc., as key properties (Cleland and Chyba 2002). Given that the search for life will ultimately require objective criteria to assess evidence for life, new, quantitative approaches to understanding *and measuring* whether or not a system is alive, and if so *how* alive it is are necessary. The challenge is, what do we measure?

Of the many attributes that could provide a foundation for quantitative approaches to characterizing living matter, the capacity of living systems to store and process information holds potential to be its most fundamental and distinctive property (Szathmáry 1989; Küppers 1990; Yockey 2005; Davies and Walker 2016). Life’s ability to structure matter and make it functional via manipulation of information is very unlike what we see in any other kind of physical system. While this view is gaining increasing traction in a variety of communities, it remains to be proven. What measurables can we assign to living systems? Will they be information-theoretic? Based on the network structure underlying information flows? Or a new formalism entirely? To address these questions it is important to start reframing the question of what life is with the tools we have at hand currently, even if these may eventually be overtaken by mathematical formalism more specific to the problem of quantifying life as iteration between theory and experiments progress. Any theory developed should have testable consequences, with relevant observables clearly defined. Among the most promising mathematical tools are those of information theory and network theory, and more recent extensions of information theory that also permit the assessment of causal interactions. So far, no other approach to characterizing living processes universally affords as promising an opportunity to quantify living processes.

Any scientific approach making progress in a field as difficult as quantifying life and its properties will have its notable criticisms and challenges. Here, one could object to an approach focused on life’s informational aspects by arguing it is too broad and ill-defined, encompassing not only processes happening within cells but also potentially those in societies or cities. The conclusions we can draw from this approach are also limited by what quantities we decide to measure, and it may be that there is not a unique solution to this problem (Davies and Walker 2016). We see both of these aspects as advantages, rather than disadvantages. There is no reason to suppose “life” is a phenomenon that happens only in chemistry, as is frequently assumed in the astrobiology community, but less so in artificial life or complex systems approaches. We instead suggest chemistry is relevant, because it is the scale of physics where life emerges, but that life itself is a broader phenomenon recurring across different scales, from chemistry to cells to societies–which more generally concerns the interactions of information (an abstract property) with matter. If true, this affords us the opportunity, even within the limited sample available to us of only one biosphere, to study many examples of life across different scales (Walker 2017). We expect this universal approach to shed light not only on the structure of life across diverse systems and scales on Earth, but also into what other living systems could be possible—that is, what other physical systems could support the same informational structures as known examples of life.

**Fig. 1:**
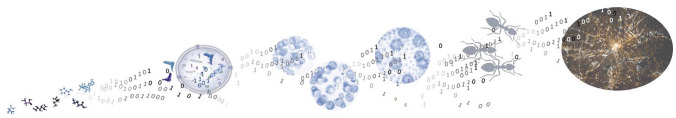
Information across the life spectrum. The figure illustrates a conceptual framework for how living systems might be considered as a gradation of examples of the same physical phenomenon with the key difference being the structure of information. It remains an open question whether an ordering of living systems on such a scale is possible, and if so what measures would characterize the scale. The complex systems in the figure represent a set of abiotic chemical compounds, the biochemical interactions within a cell, Volvox colonies composed of up to 50,000 cells, group behavior of ants’ colonies, and a social structure embedded in a city—these are not comprehensive but illustrate how such scaling of systems representing different degrees of “aliveness” might look

In this manuscript, we put forward the idea that there may be no clear black-or-white distinction between systems that are alive and systems that are not. That is, we believe there is no abrupt boundary between non-life and life, and furthermore that focusing on life/non-life as a binary distinction has been hindering progress. Instead, there could exist a gradation of states along a “life spectrum,” see Fig. 1, with some systems that are more alive than others even with similar degree of complexity. To measure the “aliveness,” information might be a good measure since it is scale-independent. Also the transition of collective systems to more alive ones may require information. For example, such measures might focus on the transformations that are possible in a given physical system, where systems that are more “alive” permit more possible transformations and often promote more improbable transformation. An example is how farming activity with leveraging the information about agriculture transformed our own civilization, leading to capabilities to transform matter that were not present before this innovation, or how humanities invention of science led to the capacity for physical systems to be launched from Earth into space, which was not possible before knowledge of the law of gravitation (Walker 2016). In this regard, life can be said to be exploring the adjacent possible (Kauffman 2019) of things that can be caused to happen. The number of transformations possible is deeply connected to the informational structure of living matter, because it is only when living systems acquire information about the physical world that they can control processes associated with that information: more information means more degrees of freedom to control.

The idea that life could exist on a spectrum is a natural consequence of an informational approach: if some concept of information really does underlie living matter as its most fundamental property, then life should be quantified in terms of its informational and causal structure (Walker and Davies 2013). Informational and causal structures, however, are properties that encompass life and extend to purely physical systems opening the possibility of unifying characterization of living and non-living systems and quantifying their similarities as well as their differences. In much the way we acknowledge that gravitation is a universal property of our universe, we should also recognize information is. And, as we look to massive objects such as planets, stars, galaxies and black holes to study gravitational physics, we should look to living processes as the most prominent examples of the physics of information we can study.

Importantly, any attempt to quantify life must deal with the cases where non-living systems can mimic the behavior of living ones. We therefore focus on collective behaviors in this manuscript, as collective behavior is widely regarded as a hallmark property of life, but is also observed across a host of non-living systems. Indeed simple physical models are often invoked to model living collective behavior, begging the question of whether or not there is a fundamental difference. Our proposal is that by studying information flows in collectives we will gain insights into determining whether information theory is capable of distinguishing non-living and living systems: if two systems, one alive and one not, display the same aggregate behavior through different mechanisms, information theory may be able to pick up on those differences. Because information theory is concerned with capturing something about the structure of correlations in space and time it is possible to use information-theoretic measures to determine whether or not two systems use the same rule set. Our hypothesis is that because life actively controls its own state via manipulation of correlations (e.g., by intervening on its own state), whereas non-life does not, information theory will be a useful tool for discerning differences in the structure of correlations in non-living and living collectives. In this regard, the spectrum of living processes may be determined by the combination of control (causation) and informational structure, with the combination quantifying entities along the spectrum from less alive to more alive. While the precise metrics and scale for the “life spectrum” remain to be defined, we herein outline some key ideas that may be useful moving forward.

## Uncovering the physics of life with information theory

Information is an abstract concept. While the mathematical theory of information, as pioneered by Claude Shannon, formalizes some aspects (described below), it is not a complete account of what information is. In particular, we do not yet have a concrete framework for understanding what information is to physics. Information can be copied between different physical media, meaning it is not strictly a material property tied only to certain physical materials. For example, information can be copied from the author’s minds to this page of text (as written on a computer), or to printed paper, and ultimately per its intended function also copied to the brain of the reader (i.e., you as you read this). These events are separated in space and time and occur across diverse physical substrates. Like many examples of the transmission of information, this suggests information cannot be a property solely of the wet chemistry of a brain, or the processing chip in a computer. It is in this sense that information is “abstract” (one might similarly consider “energy” to be abstract in the sense that it can flow between different physical systems and be stored or used in different ways, e.g., chemical energy, mechanical energy, etc., but by contrast we do have a pretty clear idea of what energy is physically).

Yet, information is also necessarily physical. In order for information to exist it must be instantiated in physical degrees of freedom (Landauer et al. 1991), and as such the dynamics of information depends on the underlying dynamics of the physical degrees of freedom. In biology, information takes on even more prominent a role, where it appears to take on “a life of its own” (Davies and Walker 2016), with explanations of biological processes suggestive that “information” itself has causal efficacy (Davies 2011). The striking ability with which life can store and process information is a subject of central importance not only for understanding the origins of life itself (Yockey 2005; Walker and Davies 2013), but also for understanding how living systems organize across the spatial and temporal scales at which we observe them. For example, information transfer and information processing are routinely called out as the driving forces behind collective behaviors such as the house-hunting behavior of ants and bees, the marvellous motions of starling flocks and fish schools, lane formation in human crowds, and so on down to the level of populations of cells (Franks et al. 2002; Couzin 2009; Deisboeck and Couzin 2009; Moussaid et al. 2009; Couzin et al. 2011). However, information is generally defined informally, often in terms of specific physical quantities characteristic of studied organisms, with a focus on the behavioral mechanisms affecting these quantities that have been largely investigated both experimentally and analytically.

### Formal definitions in information theory

To move beyond a purely descriptive analysis, or specific case studies, it is necessary to introduce methods for formalizing information content and flow. While the properties of matter have been quantified for centuries, information theory is a relatively recent development. It began in earnest with the seminal work of Claude Shannon in which a basic quantity in information theory termed entropy, *H*(*x*) is defined as (Shannon 1948):

1 $$\begin{aligned} H(X) = -\sum _{x} p(x) \log p(x) \end{aligned}$$

where *p*(*x*) is the probability for the random variable *X* to be in state *x*. *H*(*X*) is maximized when *p*(*x*) is a uniform distribution over all possible states. Equation 1 is frequently referred to as the Shannon entropy. The Shannon entropy is often described as the degree of surprise you might experience at learning the outcome of an event: the more unlikely the event, the more surprised you feel at learning about it. Viewed in this light, the concept of information is closely related to reduction in uncertainty, because the less uncertain an event is, the less surprised you are by the outcome of the event and the less information it contains. Shannon entropy forms the foundation of information theory as most measures of information are variant on it and based on the general concepts of information developed by Shannon.

**Fig. 2:**
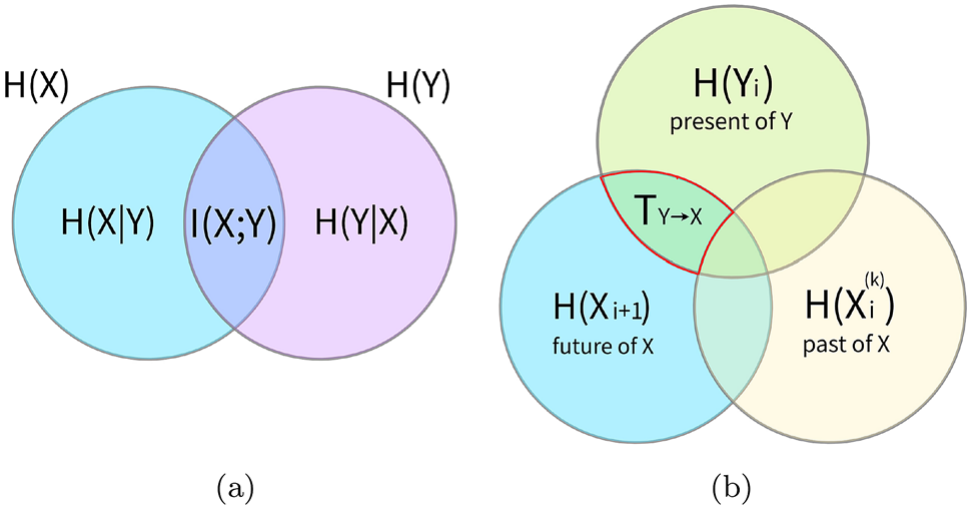
Venn diagrams illustrating **a** mutual information and **b** transfer entropy

Entropy alone, however, does not fully capture the notion of “information” which requires both a sender and receiver (Adami 2016). In this regard, information is defined as the reduction in uncertainty about a stochastic process given knowledge of other processes and is represented by the amount of shared entropy between all considered processes (Cover and Thomas 2005). This is quantified by the mutual information defined as

2 $$\begin{aligned} I(X;Y) = \sum _{x, y} p(x, y) \log \frac{p(x,y)}{p(x)p(y)} \end{aligned}$$

The mutual information can be conveniently visualized in terms of its Venn diagram as the area where the entropy of each process overlaps with each other (see Fig. 2a for an example with two processes). Consider the entropy of a particular process and remove the mutual information with all the others, what we obtain is the conditional entropy: the amount of uncertainty left about that process when we take into account knowledge of all other processes. Mutual information measures therefore the information we gain about a process from knowledge of all the others.

The mutual information measures have also been employed to quantify the amount of information stored in a system and/or in its individual components. The most popular measures used for this purpose have been *excess entropy* and *active information*. Excess entropy (Crutchfield and Feldman 2003), which is defined as the mutual information between the past and the future of a process, measures the amount of uncertainty in the future of a certain process that can be explained by looking at its past behavior. A similar measure is provided by active information, *A*(*X*). Instead of considering the entire future of the process, active information focuses only on predicting the next state that will be visited by that process (Lizier et al. 2012a).

3 $$\begin{aligned} A(X) = \lim _{k \rightarrow \infty } \sum _{x^{(k)}_n, x_{n+1}} p(x^{(k)}_n, x_{n+1}) \log \frac{p(x^{(k)}_n, x_{n+1})}{p(x^{(k)}_n)p(x_{n+1})} \end{aligned}$$

where $x^{(k)}_n$ represents past *k* states of *X* from the time step *n*, $\{x_{n-k+1},\dots ,x_{n-1},x_n \}$.

Although mutual information allows us to define and quantify information, it is a symmetric quantity and, by itself, does not capture directional relationships between stochastic processes (e.g., information transfer). To overcome this limitation and quantify information processing, Schreiber (2000) introduced the concept of transfer entropy, a measure of the reduction in uncertainty about the future state of a stochastic process given knowledge of its past (i.e., its *history*) and of the present state of one or more other processes (Schreiber 2000; Kaiser and Schreiber 2002).

4 $$\begin{aligned} T_{Y \rightarrow X} = \lim _{k \rightarrow \infty } \sum _{x_{n+1}, x^{(k)}_n, y_n} p(x_{n+1}, x^{(k)}_n, y_n) \log \frac{p(x_{n+1}|x^{(k)}_n, y_n)}{p(x_{n+1}| x^{(k)}_n)} \end{aligned}$$

As illustrated by its Venn diagram (see Fig. 2), transfer entropy leverages upon time directionality to overcome the limitations arising from the symmetry of (in this case, conditional) mutual information. Transfer entropy is a measure of directed information transfer between two or more processes in terms of predictive information (Lizier and Prokopenko 2010), i.e., it does not necessarily imply a causal interaction (James et al. 2016); its point-wise (or local) variant can provide useful insights into the spatiotemporal dynamics of collective behaviors (Lizier et al. 2008b).

## What is information theory measuring?

There exists a longstanding debate about the role of information in biological processes and whether or not biology requires a formalization of a different kind of information then what is captured in modern information theory, usually referred to as functional or semantic information [see, e.g., Yockey (2005) and Godfrey-Smith and Sterelny (2007)]. It is our view that information theory as currently formulated, particularly more recent developments in measurements that account for distributed correlation (Schreiber 2000) and causal information (Hoel et al. 2013; Ay and Polani 2008), is sufficient to make headway on addressing how information structures living matter. Specifically, our proposal is that what most distinguishes living systems is their causal and informational architecture (see, e.g., Walker (2017)) and that modern information measures, such as transfer entropy (Schreiber 2000), causal information flow (Ay and Polani 2008), effective information (Hoel et al. 2013), causal specificity (Griffiths et al. 2015), integrated information (Oizumi et al. 2014), and integrated spatiotemporal patterns (Polani et al. 2016) to name just a few, all hold promise to provide insights into the diversity and richness of biological processes from an information-theoretic perspective thus allowing windows into how we can characterize the informational and causal structure of life across a variety of spatial and temporal scales.

Velocity and directional information, for example, are physical quantities often studied in the context of coordinated motion of fish schools (Handegard et al. 2012) and starling flocks (Cavagna et al. 2010). The propagation of this type of information across a collective has also been modeled with tools from statistical physics (Bialek et al. 2012). A variety of behavioral mechanisms [for example, quorum response, a mechanism adopted by many animal species (Pratt et al. 2002; Ward et al. 2008; Sumpter and Pratt 2009)] have been investigated in similar contexts as they allow individuals in the collective to create feedback loops that amplify and dampen the transfer of information (Couzin 2009). Although formal approaches to the study of information transfer, storage and processing in these collectives are less developed (Dall et al. 2005), the large body of research focused on these phenomena provides us with strong grounds for the application of formal methods and the development of new theories. In what follows, we do not advocate the position that there is one “right information measure” for quantifying living collective behaviors, but rather that by applying diverse measures across diverse biological data sets, with appropriate controls, we will be able to start to resolve how biological systems implement manipulation of causal and correlational structure in space and time to execute function. That is we will start resolving the informational architecture of living systems that distinguishes them from non-living ones (see, e.g., Walker et al. 2016) and hopefully be able to use those insights to construct new theories that might explain what life is.

### Informational architecture

Formally, in the sense that we aim to put forward in this section the informational structure of collective behaviors (and life as pertinent example of interest) is relatively unexplored and open ground for research. Nonetheless, several studies have outlined some of its salient features. Danchin et al. (2004), for example, organize the information available to an individual in *personal information*, if it is acquired by means of direct interaction with the environment, and *social information*, if it acquired instead thanks to the behavior of other peers. They further distinguish social information between signaling and indirect observation of social cues or public information. Signaling is information transferred intentionally while social cues (e.g., the location of a forager reveals the presence of a food source) and public information (e.g., the performance of a forager provides information about the quality of a food source) lack this aspect. In the context of collective behavior, Moussaid et al. (2009) emphasize the distinction between *direct* and *indirect information transfer*. In the parlance of Danchin et al. (2004), direct information transfer corresponds to explicit signaling as well as indirect observation of social cues and public information. Indirect information transfer is instead a subset of personal information (Danchin et al. 2004) as it only includes information transferred between individuals that is *mediated through the environment*. Indirect information transfer is more popularly known as *stigmergy* and entails a form of communication in which the environment functions as a *shared blackboard* which individuals can modify to *write* information as well as sense to *read* information (Grassé 1959). The most prominent example of indirect information transfer is, undoubtedly, the pheromone-laying and -following behavior of certain species of ants (Goss et al. 1989; Hölldobler and Wilson 1990).

The above definitions, however, are primarily concerned with possible different ways in which individuals can acquire and/or transfer information. A formal framework to quantify the informational structure of collective behavior should instead account also for other essential aspects of information processing. Indeed, the intrinsic computation of a collective system is not only the result of information transfer between its components but also likely requires them to have the ability to store and transform information (Crutchfield 1994; Feldman et al. 2008). Some of these features may be subject to debate. For example, it is unclear in what sense collective chemotaxis could require individuals to store information beyond their location, but may require them only to react—however their reactions can be considered to be among a stored set of responses programmed by evolution. A formal framework of the informational structure of collective behavior could therefore include all these elements and focus on their interactions and evolution over time. A first step in this direction is to embrace a common language independent of the application domain and that affords us the possibility to formally model all aspects of information processing. This common language is provided by the theory of information.

### Measuring the informational architecture of complex systems

Information-theoretic measures were first used to study collective behaviors not too long after their introduction: to infer the amount of directional information about a food source encoded by a waggle dance in honey bees (Haldane and Spurway 1954) and by a pheromone trail in ants (Wilson 1962). Successive applications of information theory to study collective phenomena, however, have been rather sporadic (Dall et al. 2005). Mutual information, for example, has been used in statistical physics to study phase transitions in the 2-dimensional Ising model (Matsuda et al. 1996; Gu et al. 2007), in simulated flocking of self-propelled particles driven by the Vicsek model^1^ Wicks et al. (2007)) as well as in random Boolean networks of regulatory models (Ribeiro et al. 2008). In all these cases, mutual information peaks in the disordered phase or in close proximity of a phase transition. Mutual information as well as block-entropy, a variant of entropy measuring the uncertainty of finite blocks of consecutive events (Shannon 1948), have been used to study collective decisions in simulations of pheromone-laying ants (Klyubin et al. 2004) and to show in this context that a limited amount of noise is beneficial to information transfer (Meyer 2017). More recently, Gelblum et al. (2015) showed that ants that attach to a collectively transported load inject information into the system and that this information is effective only for a short period of time. These measures have been used, not only as analytical tools, but also to artificially evolved controllers to design collective motion behaviors for modular (Prokopenko et al. 2006) and multi-robot systems (Sperati et al. 2008).

Information theory deals with the quantification of information, its storage and transfer (Cover and Thomas 2005) in a large number of different scientific and technological domains. In the context of biology, for example, information theory is extensively used to study the functioning of the nervous system and to determine its structure (Honey et al. 2007; Vakorin et al. 2009; Nigam et al. 2016; Ito et al. 2011; Lizier et al. 2011; Vicente et al. 2011). Active information (see Equation has been used, among other examples, to study information storage in cellular automata (Lizier et al. 2012b), neural information processing (Wibral et al. 2014) as well as swarming dynamics (Wang et al. 2012) while excess entropy was primarily applied to the study of complex physical phenomena (Crutchfield and Feldman 2003).

While the application of other measures of information flow and causal information will additionally provide insights into the issues we discuss, here we focus primarily on the transfer entropy as a measure of information processing. We note our choice of transfer entropy provides an illustrative example, motivated by the application of transfer entropy from a large number of different domains including neuroscience and finance (Bossomaier et al. 2016) As for mutual information, transfer entropy has been used to study information flow in different phases of a 2D Ising model (Barnett et al. 2013). Lizier et al. (2008b) used it to study cellular automata and to show that particles (defined as gliders and domain walls) are the main means of information transfer in this type of model. Wang et al. (2012) investigated information cascades in simulations of collective motion by artificial particles showing that they take the form of waves rippling across the swarm. Other collective behaviors that were studied in simulation using the framework of transfer entropy include the dynamics of regulatory networks of the yeast cell-cycle (Kim et al. 2015; Walker et al. 2016) (discussed below) and consensus achievement in multi-agent systems (Valentini et al. 2018). It has also seen some moderate application in the study of collective animal behavior. For example, to study information transfer underlying schooling of pairs and small groups of zebrafish providing a useful tool to identify leadership relations (Butail et al. 2014, 2016; Mwaffo et al. 2017) as well as informative and misinformative interactions during fish U-turns (Crosato et al. 2018); to study leader-follower relationships in bats (Orange and Abaid 2015) as well as ants and termites (Valentini et al. 2020); information transfer in slime molds (Ray et al. 2019); and predator-prey interactions between pairs of fish (Hu et al. 2015).

As evidenced by the breadth of measures and their widespread applicability, there is no clear consensus on the best unified approach to applying information-theoretic insights across diverse systems, or even whether such an approach is possible. Each measure provides a projection of the causal and correlational structure of a dynamical system into a lower dimensionality and therefore does not capture the full picture. Together different measures can provide insights into the structure of living and non-living systems, which in turn can be leveraged to take the next steps developing new theories and measures for what life is. Thus, there is promise for informational approaches to understanding diverse forms of life, but much work needs to be done to build a unified framework.

### An example: informational architecture across collectives with similar behavior but different mechanisms

Collective systems often face the need to make decisions that are commonly agreed upon at a group level. Such decisions are made by large numbers of agents that follow simple interaction mechanisms to gather, transfer, and process the information necessary for a collective decision. Information transfer during collective decision making is of paramount importance: without the exchange of information, no consensus among the collective can be achieved. Despite this and of our currently good understanding of the mechanisms of collective decision, little is known about the contribution of individual rules in determining the transfer of information across the group leading to a decision.

To address this issue and better understand the information profile of different decision-making mechanisms, we studied the landscape of information transfer originating from different decision-making strategies applied to the same decision problem (Valentini et al. 2018): the achievement of a consensus on the higher-valued of two options (Valentini et al. 2017). We considered two decision mechanisms, the majority-rule and the voter model, that are used by agents in a collective of 100 to change their opinions. In our model (Valentini et al. 2016), agents always have a preference for either of the two options, *A* with quality 1, and *B* with quality $\in \{0.5, 0.9\}$. They alternate between a period of exploration, in which each agent travels to a region of the environment associated with its current option in order to sample the quality of this option, and a period of dissemination proportional to the estimated quality in which the agent locally broadcasts its opinion to other agents with a common area. Between dissemination and an exploration period, agents apply a decision mechanism and possibly change opinion. When using the majority rule, the agent switches opinion to the one favored by the majority of its neighbors (Valentini et al. 2015). When using the voter model, an agent simply adopts the opinion of a neighbor chosen at random (Valentini et al. 2014). In both cases, we considered a neighborhood composition that varies over time according to motion dynamics of the agents but is of fixed cardinality with always 5 neighbors.

**Fig. 3:**
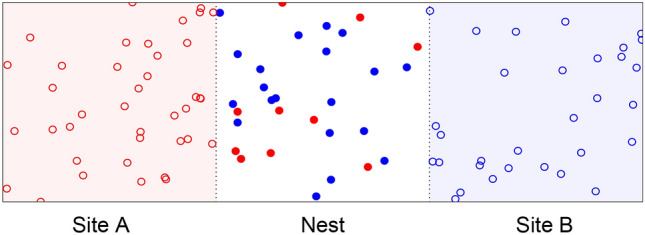
Illustration of the simulation environment partitioned into site *A* (red area), nest (white area), and site *B* (blue area). Symbols: filled circles represent agents in the dissemination state, empty circles represents agents in the exploration state, colors represent agent’s opinion (red for site *A*, blue for site *B*). Image reproduced from Valentini et al. (2018) (color figure online)

**Fig. 4:**
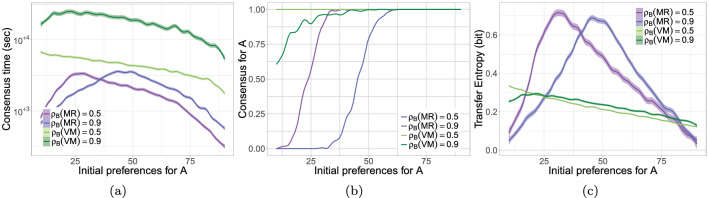
**a** Consensus time (logarithmic scale), **b** proportion of simulations converged on consensus for site *A*, and **c** transfer entropy over the initial number of agents favoring site *A* (i.e., $\{10, 11, \ldots , 90\}$) for a collective of $N=100$ agents. Problem configurations: majority rule $\rho _B(MR) \in \{0.5, 0.9\}$, voter model $\rho _B(VM) \in \{0.5, 0.9\}$. Figures report the estimate of the smoothed conditional means and their confidence interval computed using LOESS regression with a span of 0.1 of the data. Images reproduced from Valentini et al. (2018)

The mechanistic differences between the majority rule and the voter model lead to different performances: the majority is faster (see Fig. 4a) but less accurate (see Fig. 4b) than the voter model (cf. Valentini et al. (2016)) for a deeper discussion of this result). On this basis, we can interpret the flow of information that an agent applying a decision rule (i.e., either the majority rule or the voter model) receives from its surrounding neighbors. We did so by applying transfer entropy to data gathered from spatial multi-agent simulations (see Fig. 3). We found that, as for speed and accuracy, the information transferred among agents is dependent on the decision mechanism. The amount of information transferred from neighbors to a focal agent applying a decision rule increases with the time necessary for the collective to achieve a consensus decision and is loosely modulated by the uncertainty of the final outcome. This example highlights the relationship between informational structure and local rules, even in a situation where the collective behavior might ultimately lead to similar decision-making. It highlights that informational structure is not merely a property of the decision to be made, but a property of how the microscopic dynamics and macro-scale features are related via the structure of correlations among component parts.

To further illustrate this point, we can use recent work by some of us comparing the structure of information flows of the tandem run behavior of *Temnothorax* ants with those of two species of termites, *Reticulitermes speratus* and *Coptotermes formosanus* (Valentini et al. 2020). We focused on the tandem running behavior of *Temnothorax* ants, and used transfer entropy to study information flows between leader and follower ants. Tandem runs allow ants informed about potential sites (leaders) to lead uninformed ants (followers) about their location. Tandem runs proceed in bouts of small, straight segments by the leader and more variable movements by the follower (perhaps while memorizing landmarks (Bowens et al. 2013), punctuated by physical contact with the leader by the follower which triggers a repeat of this sequence. This bidirectional feedback (i.e., the follower follows and leader waits) has been postulated to implement a learning process akin to teaching in humans. In contrast to *Temnothorax* ants, the purpose of termites’ tandem runs is to ensure that newly formed couples of female and male termites do not lose each other while exploring their environment in search of a new nest site. The leader of a tandem run in termites is not informed about a potential nest site and therefore is not attempting to communicate such information to the follower. We analyzed the magnitude and direction of information exchange between tandem pairs using transfer entropy. Three communication channels were considered: motion, rotation, and combined motion-rotation. The space-continuous trajectories of tandem pairs were discretized according to different encoding schemes based on the channel in question:

- motion channel: moving or not-moving
- rotation channel: clockwise or counter-clockwise
- motion-rotation channel: not-moving, moving clockwise or moving counter-clockwise

We identified previously unknown and striking differences between information exchanged between leader and follower in the tandem run behavior of ants and termites. While in termites the leader of a tandem run is in control of all aspects of the pair’s behavior (i.e., both motion and rotation), this is not the case for *Temnothorax* ants. Contrary to current views, our analysis revealed the leader and the follower ants *both* play different “leading” roles. The directionality of information flow shows the leader directing the rotation pattern of the couple, but it is the follower who determines the motion pattern of the pair. In this manner, the leader-follower pair in *Temnothorax* exchanges different information over different channels, and the pair's interactions encode a different directionality for information flow in different degrees of freedom. This highlights an explicit example where it is necessary to share information to make collective decisions. Rigorous quantification of information flows in collective systems has the potential to provide new insights into how new kinds of computation (e.g., decision-making) can emerge from the actions of individual agents because of subtle differences in the micro- and macro-level behaviors.

## Characteristics of the global causal structure of collective systems

Diverse approaches have been employed to characterize the causal structure of complex systems, especially biological systems, and it is often argued at least some of the properties of the causal structure might be essential to the operation of living processes (Ellis et al. 2011; Griffiths et al. 2015; Auletta et al. 2008; Davies 2011; Roederer 2006). To distinguish the collective behavior of living systems from non-living systems, it will likely be necessary to understand the relationship between the informational architecture and these characteristics. Here, we propose what enables differentiating the collective behavior of living systems from other complex physical systems is how informational architecture and its physical instantiation, that is the causal structure underlying the computation, are coupled. In the last section we saw an example of how informational structure was changed by changing local interaction rules. Here by focusing on causal structure, what we mean is the global structure of the state transition diagram, which is related to the function of a system. In this regard, there are multiple microscopic realizations possible for the same “behavior” (causal structure of the state transition diagram) [see, e.g., discussion in Hanson and Walker (2019)].

In this section, we review previous studies on the emergent properties of the causal structure of complex systems such as criticality, controllability, and causal emergence along with studies indicating their relationship with the information processed by biological systems. Our goal is to specifically discuss this work in relation to the role of causal structure in architecting information flows unique to biological processes, and thereby taking steps toward quantifying life. For more extensive reviews on complex systems and the specific topics we touch on here, the reader is referred to more general perspectives (Munoz 2018; Liu and Barabási 2016; Oizumi et al. 2014).

### Criticality

Complex systems often exhibit multiple types of collective behaviors, called phases, that are distinctively, often drastically, different from each other and from behaviors of individual entities composing the systems. When phase transitions occur the global states can be changed from one phase to a different phase by even a tiny local perturbation on a system (Anderson 1972; Stanley 1971; Chaikin and Lubensky 2000). A large body of literature hypothesizes biological states might be associated with a particular class of phases, with the implication dynamical changes in biological states or the emergence of biological states from non-living (random) collective states could be characterized as phase transitions (Anderson 1972; Hopfield 1994; Pollack and Clegg 2008). Here, we focus on the phase transition between two different dynamical regimes widely discussed in the literature: stable and chaotic, and critical systems balanced between these two regimes. A wide variety of biological systems from neural firing to cellular gene regulation and animal motion are reported to be critical (Beggs 2007; Haldeman and Beggs 2005; Mora and Bialek 2011). It is observed that compared to systems near their critical points, systems far from criticality are either too stable to be adaptive in the ordered phase or too unstable to be robust in the chaotic phase. Hence, the tendency of the living systems is to tune (e.g., via evolutionary selection) to critical points, such that the critical dynamical regime is suggested as a driving factor for homeostasis and evolvability, hallmarks of fundamental biological processes (Kauffman 1993).

Moreover, the computational capability of complex systems, defined as the amount and diversity of mappings between inputs and outputs via the internal logic structure to perform certain tasks, is conjectured to be optimized at the critical phase (Turing 1950; Crutchfield and Young 1988; Packard 1988). This conjecture was formulated as a problem related to the operation of information supporting computation, and it has been suggested that computation occurs more naturally near critical points (Langton 1990) possibly with information storage, propagation and processing capabilities maximized (Kauffman 1993). Furthermore, Mitchell (2006) emphasized the importance of understanding information dynamics through time series of dynamic states in networked systems. Subsequently, several other studies reported results indicating information processing in complex networks is maximized in their critical phase (Solé and Valverde 2001; Kinouchi and Copelli 2006; Ribeiro et al. 2008). Especially, Lizier et al. (2008a) seeks to improve on previous attempts to measure these computational properties, with a thorough quantitative study of the information dynamics in Random Boolean Networks (RBNs)(Kauffman 1993), widely accepted as models of Gene Regulatory Networks (GRNs). They show that averages of active information and transfer entropy quantifying information storage and processed information, respectively, reach maximum near the critical point (Lizier et al. 2008a).

There are some implications here for the limits of typical physics models, such as mean-field approximations, and renormalization group approaches, which may not always translate to biology because of the differences in collective behavior across living and non-living systems [e.g., the transition to life might be a discontinuous transition in informational properties, see Walker and Davies (2013)]. That is, in biology it is unclear if mean-field approximations will in general work, and if theories that renormalization group approaches are typically applied to continuous phase transitions could be useful to the discontinuities that may characterize biological phase transitions. One path to a solution is to study distinctive characteristics of critical systems related to biological functions, and how these characteristics manifest biological networks. For example, Daniels et al. (2018) recently demonstrated 67 gene-regulatory networks are critical, whereas random ensembles with similar causal and informational architecture were shown not to be, showing how the specific causal and informational properties of functional, biological networks can be isolated by their critical properties. Criticality has even been shown to be a selectable property in random gene network models (Serra 2019). Other recent studies by our group and others have highlighted the balance between adaptability and robustness in causal interactions with the environment and in internal information processing (Walker et al. 2016), and the characteristics of information processing are known to exhibit a strong dependence on distance from the critical transition between the stable phase (dominated by information storage) and the chaotic phase (dominated by information transfer) (Lizier et al. 2008a; Kim et al. 2015). Critical systems are known to possess two essential characteristics of biological systems: adaptability for and robustness against a wide range of varying environmental conditions. We expect that the ubiquity of criticality in gene regulation will inform related studies on the unique information processing and control properties of biological networks.

### Control kernel

One of the main interests in complex systems research is to understand, and ideally predict, emergent global dynamics such as the trajectories or final states of a system (Liu and Barabási 2016). These global dynamical states representing the collective behavior of systems can be altered by external causal intervention. Steering a whole system from its initial state to a desired final state via external causal intervention provides a powerful tool for numerous applications from problems related to opinion dynamics, to epidemics, cell differentiation, and social insects’ nest choice, to name a few. Recent research developing the mathematical foundations for controlling complex networks seeks to find rigorous control mechanisms by understanding causal structure and/or the updating rules for dynamical states of individual components. Liu et al. (2011) developed their pioneer framework to reduce the problem of controllability of directed complex networks with linear dynamic process to a graph-theoretical problem. Using their theory, they identified a set of driver nodes that can fully control the dynamics of a whole network. They also found that the identified driver nodes tend to be low-degree nodes and the total number of driver nodes is determined mainly by the degree distribution of the network.

Most models for biological systems are nonlinear dynamical systems and full controllability is neither feasible nor necessary. Instead, it is more realistic and often sufficient to find a specific control mechanism able to steer a given system from a particular initial state or trajectory to a certain set of desired states or trajectories (Cornelius et al. 2013). For example, one of the main purposes of controlling gene regulatory networks is to steer the system to particular cellular states rather than any arbitrary expression level. This phenotypic control is often localized in a small fraction of the entire network, defined in terms of a control kernel that is able to drive the system to a desired attractor. Yet, the size of the control kernel is larger than one for random networks in general (Liu et al. 2011; Kim et al. 2013; Gates and Rocha 2016; Choo et al. 2018). This suggests that there might be a certain range of degree of controllability that can be quantified by the size of the control kernel for biological systems. It is for this reason that we view the degree of controllability as a fundamental characteristics of the informational architecture of biological systems (Walker et al. 2016; Kim et al. 2015), which distinguishes living collectives from all non-collectives.

### Causal emergence

Identifying the causal relationships within complex systems is one of the most fundamental ways to understand their emergent behaviors. However, it is a difficult task: not only is causal structure complicated in complex systems (because they are, frankly, complex), but also because the causal structure can be analyzed at multiple spatiotemporal scales, and it is often not clear which scale is preferred (if any) for analysis. For a long time, it has been believed that the principal causal structure can be defined only at the lowest (micro) scale and causal structures at higher scales are useful but merely crude descriptions of the principal one. In other words, it is assumed micro-level causal relationships fix all higher level causal structures and there is no actual causal contribution from higher (macro) level causal analysis (Kim 1993, 2000). This reductionist approach has been widespread success across various disciplines of science, including major developments in physics and chemistry. Nonetheless, the possibility for causal emergence, where macro-scales are important drivers of casual structure, is raised again and again as providing simple explanations for various types of biological collective behaviors, from epigenetics to collective decisions by ant colonies, to mental states in brains to name a few. While most previous studies about causal emergence have been limited to qualitative arguments, recently quantitative research done by Hoel et al. (2013) and Hoel (2017) has shown that it is possible that in some systems causal structure cannot be completely captured by the micro-scale structure, because the macro-level causal structure is more effective and informative. We here suggest that finding the control mechanism of a complex system (as described in the previous section) can be a useful tool to investigate different levels of causal structure since it provides insights about the local (micro) level causal influence from driver nodes on the global (macro) level effect as the steered dynamical states of the whole system.

### Coupling between informational architecture and causal structure of biological collective systems

As discussed in the previous section, relationships between informational architecture and causal structure of living systems are implied in some previous studies. However, it still remains vague and inconclusive whether there is a meaningful relationship between informational and causal architecture, and if so, whether that relationship can distinguish living collective behavior. Here we provide a quantitative example of this approach, building a dynamical model for biological networks based on empirical datasets whose small size allows statistical analysis. Boolean network models for cellular biological pathways are one of the best candidates satisfying this necessary condition, due to the advent of high-throughput technologies and data driven approach in Systems Biology. Recently, Daniels et al. (2018) provided the most comprehensive survey of criticality to date, studying it across 67 cellular regulatory Boolean networks obtained from the Cell Collective database (Helikar et al. 2012), showing all of the networks are near critical. On the other hand, Kim et al. (2013) analyzed various biomolecular regulatory Boolean networks and identified control kernels, a minimal set of nodes necessary to be regulated to control the cellular network to reach a desired state. The study reveals that for most of the networks, a control kernel is only a small fraction of components of the network. Therefore, it is a natural choice for us to leverage the same kind of biomolecular regulatory Boolean networks to study if, or how, biological systems couple information structure to their causal mechanism. Here, we demonstrate how the relationships between informational architecture and causal mechanisms in biological systems (Walker et al. 2016; Kim et al. 2015) can be studied with a case study in the cell cycle process of *S. pombe*, a simple example of the central aspect of biological function.

#### Description of Fission Yeast Cell Cycle Boolean Model

**Fig. 5:**
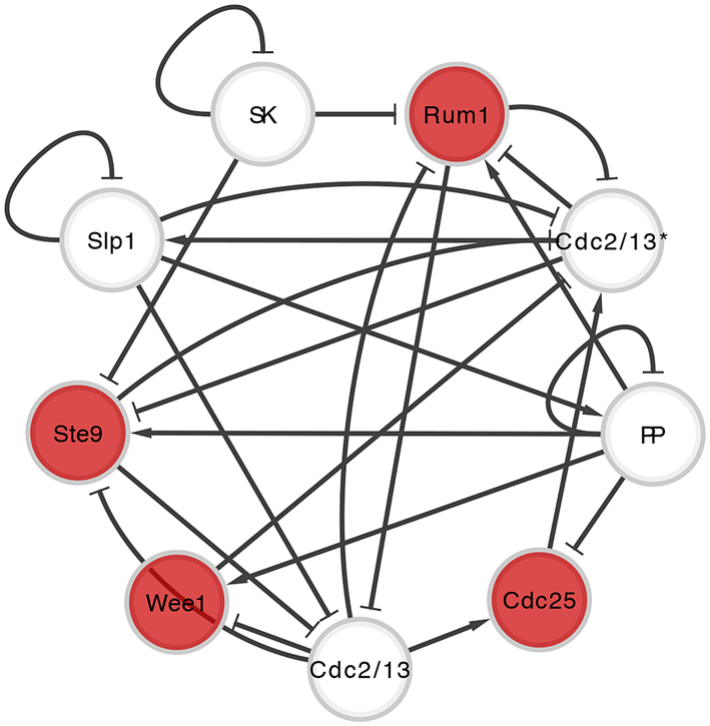
Boolean network model of fission yeast cell-cycle regulation. Nodes represent the regulatory proteins, and edges denote two types of biochemical interactions between nodes: activation (ended with an arrow) and inhibition (ended with a bar). The nodes colored red are the control kernel, which regulates the global behavior of the network when pinned to specific values. The figure is regenerated based on the data from Davidich and Bornholdt (2008) and Kim et al. (2013)

The Boolean network modeling the cell cycle of *S. pombe*, also known as fission yeast (see Fig. 5) consists of 9 key proteins (Davidich and Bornholdt 2008) that govern the cellular functions in the following steps named as G1-S-G2-M phases: from the cell growth through DNA replication to cell division into two daughter cells. In the network, each node representing protein has a Boolean value of 0 or 1, indicating its absence or presence, respectively, and the combined set of every node’s state is referred to as the network state or the network configuration. The state of an individual node is determined as a function of the states of other nodes biochemically interacting with it and the type of interaction, inhibition or activation. Applying a local dynamic rule to every node simulates one time step evolution of the system. Repeating the time evolution on the particular network configuration that corresponds to the starting point of the cell-cycle process reproduces the sequence of phases of the biological processes in an accurate order. Starting with each of the 512 possible network states generates the network state space, all possible dynamical trajectories of the time evolution whose landscape represents the global dynamics of the fission yeast cell-cycle Boolean network. The network state space includes 13 attractors, a single network state or a set of multiple states where any time evolution trajectory in the state space converges. Since the dynamics is deterministic, the entire network state space can be divided into 13 disjoint subgroups according to which attractor each network state converges to. Amongst them, the primary attractor where around 74% of network states end is the only biologically functional one (Davidich and Bornholdt 2008).

The recent study by Kim et al. (2013) on controlling these time evolution trajectories of biological regulatory Boolean networks showed there exists a control kernel, a minimal subset of nodes whose fixed values dictate the fate of the whole system by reforming the attractor landscape for all states to converge to the primary attractor. For the fission yeast cell-cycle network, it was shown that 4 proteins out of the whole networks are control kernel –that is, when the states of Rum1, Wee1, Ste9 and Cdc25 are fixed to 1, 1, 1, 0 which is the same as their states in the primary biologically functional attractor, all time evolution trajectories initiated from every possible network configuration converges to the biologically functional attractor.

#### Relationship between information transfer and control kernel: a case study of the fission yeast cell cycle network

In our recent work (Kim et al. 2015), we quantify the informational architecture of the fission cell cycle network by calculating the transfer entropy between every pair of nodes in the network, identifying biologically distinctive patterns from the results and demonstrating its close relationship with control mechanisms via the control kernel. To distinguish the informational patterns characterizing the biological system, we compare the results from the transfer entropy analysis with the same analysis performed on ensembles of random networks constructed to share certain topological features with each yeast network, but which do not execute the same biological function. For the purpose of comparison, we utilize two types of random network ensembles, Erdös-Rényi (ER) and Scale-Free (SF). The ER random networks share no structural bias with the fission yeast network other than the size of the network. The SF random network ensembles, by contrast, maintain the same number of activation and inhibition links for each individual node as the cell cycle networks, which makes SF share the same topological degree distribution with the biological network.

**Fig. 6:**
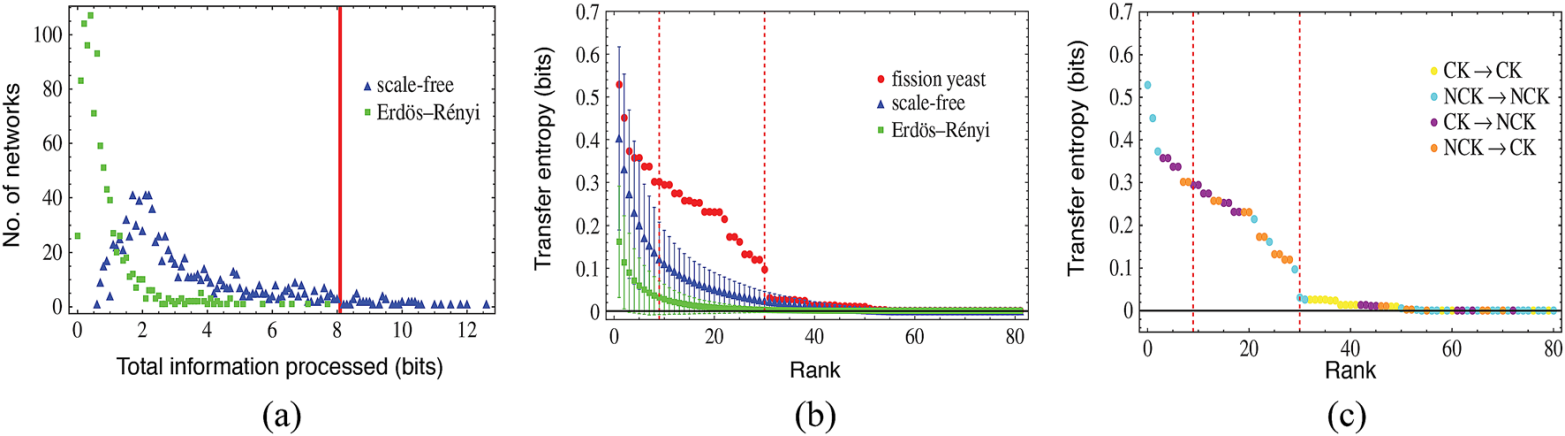
Informational architecture and causal structure within fission yeast cell cycle network. **a** Distributions for total information processed for the ensembles of ER (green) and SF (blue) networks. Each data point represents the number of individual networks within the respective ensemble with a given amount of total information transferred on *x*-axis. The red line indicates the total information processed for the fission yeast cell-cycle network. **b** Scaling of transfer entropy among pairs of nodes for the fission yeast cell-cycle, ER and SF networks represented with red, green and blue, respectively. The averages and the standard deviation for each of the random network ensembles are computed for a sample of 1000 networks. Regions between dashed lines denote the biologically distinctive rage for the information scaling for the fission yeast. **c** Scaling relations for information transfer for the fission yeast cell-cycle networks. CK represents the control kernel nodes and NCK is a set of nodes that are not included in the control kernel. Data shown are the same as the scaling patterns of the transfer entropy of the fission yeast in the Center panel. The scaling patterns are divided into four classes of information transfer depending on whether each node in a pair is in CK or NCK. The figures are adopted from Kim et al. (2015) (color figure online)

We define the total information processed within a single network as the sum of transfer entropy between all pairs of nodes in it. The results show that the yeast cell cycle network processes more information than most random networks in either ensemble (see Fig. 6a). This is a directly measured result supporting the hypothesis relating information processing and criticality, i.e., that there exists optimization of information transfer near a critical point in biological systems, where here this is shown quantitatively, unlike previous studies that are qualitative or done with general models rather than ones based on empirical datasets. We also investigate the distribution of the processed information across the individual networks. We do so by analyzing the rank scaling of every pair of nodes according to its measured value of transfer entropy. The scaling relations reveal that biological networks are significant outliers compared to random networks (see Fig. 6b). What is most biologically distinctive in informational management is that information is processed much more evenly in biological networks in contrast to random networks. The scaling pattern exhibits a statistically distinctive range of high-mid ranked pairs of nodes where transfer entropy is significantly larger for the biological network when compared to corresponding ranked nodes in the random networks. Moreover, we find that this biologically distinctive range is dominated by information processing between control kernel nodes and non-control kernel nodes (see Fig. 6c). Therefore, we find that the cell-cycle network exhibits characteristic patterns in its informational architecture setting them apart from their random network counterparts. These patterns are related to control of the global causal structure (flows in the state-transition diagram) via the control kernel nodes, which play a dominant role in information transfer within the network and regulate the global state transitions.

## Non-living physical systems

So far we have discussed mostly biological examples of collective behaviors, but to advance toward our goal of understanding unique features of living collectives, we must first understand where properties of interest appear in non-living systems. For example, non-living, purely physical systems allow us to study controllability in the absence of biological organization. Gravitating bodies, Van der Waals gases, and spin glasses are all examples of potentially complex systems that would qualify as purely physical. Unfortunately, the vast majority of these systems never undergo any sort of controllability analysis, as there is rarely a practical need to control them. The exception to this is coupled quantum systems, where the potential for quantum computing has brought intense scrutiny to their controllability.

While a full review of controllability in quantum systems falls outside the scope of this paper, it is worth pointing out a few of the more salient results and their relation to network controllability. Most notably, many coupled quantum systems allow full controllability via the manipulation of just one or two qubits, which play the role analogous to driver nodes in network control theory. A quantum system is said to be fully controllable if the entire Hilbert space is accessible by utilizing some form of external controls (Schirmer et al. 2003). Given a sufficient amount of external control, any system should be fully controllable; for example, a chain of coupled spin-1/2 particles will be trivially fully controllable if one has control over each individual spin. The more interesting question, then, is what the minimal external controls needed are in order to fully control the system. This is the same question as Liu et al. (2011) and others put forward in the context of network controllability, and the answers are surprisingly similar. Specifically, Burgarth et al. (2009) examine the conditions for full controllability of a chain of spin-1/2 particles coupled together via isotropic Heisenberg-type interactions and find that control over the state of a *single node* at the end of the chain via two non-commuting external controls is sufficient for full control over network. Similarly, in a Heisenberg-type spin chain with XY type interactions (no coupling between Z spin components), full control can be achieved by the manipulation of just a single site and its interaction with its neighbor (Schirmer et al. 2008; Kay and Pemberton-Ross 2010). Not all spin chain systems are so easily controllable, however, as Ising-type interactions, for example, require control over each local spin for full controllability (Wang et al. 2016). In fact, much like network controllability, it is difficult to tell whether a given topology and interaction-type will yield full controllability in a small number of driver nodes and much of the research being done in the field of quantum controllability is focused on addressing this question.

It is difficult to know whether the non-classical nature of quantum systems biases our understanding of control in purely physical systems. What is clear, however, is that some of these systems either allow global control via a small number of local interactions or they require manipulation of every component. This is consistent with studies of controllability of complex networks mentioned in “Control kernel” section: scale-free networks including biological networks tend to require more driver nodes for full controllability than random networks and less than completely disconnected systems, where every node needs to be manipulated. This might indicate that the characteristic degree of controllability for biological systems lies at critical regime similarly to how the dynamics of biological systems are known in many cases to critical (see “Criticality” section). Hence, in general, more extensive analysis of physical collective systems, in parallel to that of living collectives, may prove fruitful to determine precisely what features are unique to the physics of life versus being a broader property of our universe.

## Discussion

Advances in our understanding of life across all scales are converging on concepts of information as a, if not the most defining property of life (Nurse 2008). As we have partly reviewed, the tools of information theory have been used to explain diverse biological phenomena ranging from the firing of neurons in the brain (Honey et al. 2007; Vakorin et al. 2009; Ito et al. 2011; Lizier et al. 2011), to the behavior of schooling fish (Butail et al. 2014, 2016; Mwaffo et al. 2017; Crosato et al. 2018), to chemical signaling within cells (Cheong et al. 2011; Rhee et al. 2012; Selimkhanov et al. 2014). At the same time, realizing a true “physics of living systems” requires a more fundamental understanding of life than what is currently known (Bialek 2012). Among the most challenging open problems is the lack of *quantifiable* metrics distinguishing living systems from non-living ones (Cleland and Chyba 2002; Davies and Walker 2016). While much of current biology can proceed without a deep, mathematical understanding of what life is (and what it is not), there are some areas of science where objective, measurable criteria for what constitutes life are absolutely critical: these include the origins of life and the search for alien life (Walker 2017). In our efforts to find new examples of life in the lab (Cronin and Walker 2016) or on other worlds (Walker et al. 2018), quantifiable metrics will be the deciding factor—allowing the possibility to design evolvable chemical systems in the lab traversing the pathway(s) from non-life to life, or permitting unambiguous detection of life even as it might exist in radically different chemistries from those of known life.

One of the most significant hurdles in building a quantitative understanding of life is our lack of appropriate controls for isolating the physics of living processes. While we have a reasonable understanding of standard physics as it operates in life (Schrödinger 1992; Hoffmann 2012), we have so far not designed appropriate experiments to isolate whether or not a direct comparison between the attributes of living and non-living systems is even possible, or whether it would resolve differences in the collective behavior across non-living and living systems. It may be that no such distinction is meaningful. To address the need for better controls for studying the physics of living processes, we have proposed a set of ideas establishing a framework where life exists on a continuum from what we currently regard as non-living systems that might exhibit some “life-like” behaviors all the way to planetary scale civilizations. This is a natural consequence of the idea that living systems might exist on a spectrum, where some systems are more alive than others, with the key distinguishing attribute being their informational architecture. While we propose that such a scale could exist, we are not yet at the stage of establishing the proper metrics for this scale, nor placing particular examples on it. One of the challenges is that a comparative analysis across many collective systems remains to be rigorously developed and defined. We regard information theoretic approaches as the most promising because they capture the correlational and causal structure of physical systems as they exist in space and time, and provides a sufficiently abstract and rigorous mathematical framework to be a candidate for quantifying life across the wide variety of physical media and scales we find living processes. It is necessary to share information to make collective decisions: as such, rigorous quantification of information flows in collective systems is one avenue that has the potential to provide new insights into how new kinds of computation (e.g., decision-making) can emerge from the actions of individual agents.

With advances in the use of artificial intelligence and information theory, it is now possible to extract algorithms for biological behavior across many different channels for information processing in living systems, e.g., motion, morphology, and bioelectric fields. However, despite advances in both conceptual and technical application, no systematic analysis has yet been performed quantifying the informational properties of living systems by directly comparing to those of non-living ones. Most models of biology have been constructed *in silico* or in robots, meaning there is no possibility to directly compare the physics of these simulated systems to that of biology, which exists in wet, messy chemistry. This substantially limits the ability of current artificial life models to distinguish physics that merely appears “life-like” from any that might rigorously distinguish living matter. The examples in “An example: informational architecture across collectives with similar behavior but different mechanisms” section  suggest one step further, emulating the same behavior in different systems may be insufficient if it does not capture local-level rules as information flows will be different. As a consequence, in silico simulations, if naively constructed, may be inadequate for understanding the physics of information flows in real biological processes. In computers the modeler explicitly chooses the rules, and since we have shown information theory is sensitive to the particular choice of rule(s), this biases direct comparison of informational architecture in computer-simulated life as compared to real-world biological systems. In particular, it does not allow isolating what the structure of information flows might look like in living systems, above and beyond what is explicitly encoded by the laws (rules) of physics and chemistry—which we hypothesize is a critical step in understanding the physics of life (Walker and Davies 2013). Furthermore, computer models are often low-dimensional representations of real-world processes and therefore do not capture all of the multiple channels through which biological agents might process information. One path forward may be by building physical simulations of living systems. For example, the collective behavior evolved in oil droplet mixtures provides a means to *physically simulate* biological properties in a system that is not alive, rather than merely simulating in a computer (Taylor et al. 2017; Points et al. 2018). Thus, one path forward to address the questions we open here is to develop physical simulations for living systems as a means to probe the physics of information flows.

Much remains to be done to determine whether or not this approach will ultimately bear fruit; however it is our view that taking steps in this direction will nonetheless provide new insights into biology and in particular provide new quantitative tools for understanding when collective behavior can be attributed to living processes and when it cannot.

## Footnotes

1 The Vicsek model is a model where self-propelled particles, i.e., particles with infinite energy, move at constant velocity while trying to match their direction of motion with the average direction of their neighbors (Vicsek et al. 1995).
